# Association of Cardiovascular Risk Trajectory With Cognitive Decline and Incident Dementia

**DOI:** 10.1212/WNL.0000000000200255

**Published:** 2022-05-17

**Authors:** Bryn Farnsworth von Cederwald, Maria Josefsson, Anders Wåhlin, Lars Nyberg, Nina Karalija

**Affiliations:** From the Umeå Center for Functional Brain Imaging (B.F.v.C., M.J., A.W., L.N., N.K.), Department of Integrative Medical Biology (B.F.v.C., L.N.), Center for Demographic and Aging Research (M.J.), Department of Statistics (M.J.), Umeå School of Business, Economics and Statistics, and Department of Radiation Sciences, Diagnostic Radiology (L.N., N.K.) and Radiation Physics (A.W.), Umeå University, Sweden.

## Abstract

**Background and Objectives:**

Cardiovascular risk factors have a recently established association with cognitive decline and dementia, yet most studies examine this association through cross-sectional data, precluding an understanding of the longitudinal dynamics of such risk. The current study aims to explore how the ongoing trajectory of cardiovascular risk affects subsequent dementia and memory decline risk. We hypothesize that an accelerated, long-term accumulation of cardiovascular risk, as determined by the Framingham Risk Score (FRS), will be more detrimental to cognitive and dementia state outcomes than a stable cardiovascular risk.

**Methods:**

We assessed an initially healthy, community-dwelling sample recruited from the prospective cohort Betula study. Cardiovascular disease risk, as assessed by the FRS, episodic memory performance, and dementia status were measured at each 5-year time point (T) across 20 to 25 years. Analysis was performed with bayesian additive regression tree, a semiparametric machine-learning method, applied herein as a multistate survival analysis method.

**Results:**

Of the 1,244 participants, cardiovascular risk increased moderately over time in 60% of sample, with observations of an accelerated increase in 18% of individuals and minimal change in 22% of individuals. An accelerated, as opposed to a stable, cardiovascular risk trajectory predicted an increased risk of developing Alzheimer disease dementia (average risk ratio [RR] 3.3–5.7, 95% CI 2.6–17.5 at T2, 1.9–6.7 at T5) or vascular dementia (average RR 3.3–4.1, 95% CI 1.1–16.6 at T2, 1.5–7.6 at T5) and was associated with an increased risk of memory decline (average RR 1.4–1.2, 95% CI 1–1.9 at T2, 1–1.5 at T5). A stable cardiovascular risk trajectory appeared to partially mitigate Alzheimer disease dementia risk for *APOE* ε4 carriers.

**Discussion:**

The findings of the current study show that the longitudinal, cumulative trajectory of cardiovascular risk is predictive of dementia risk and associated with the emergence of memory decline. As a result, clinical practice may benefit from directing interventions at individuals with accelerating cardiovascular risk.

An estimated 40 to 50 million people currently live with dementia, a number that is expected to rise 3-fold to 152 million by 2050.^1^ Of these dementia cases, ≈60% to 80% are a result of Alzheimer disease (AD), while ≈10% are considered to result from vascular insult.^2^ Despite the extensive social and economic burden of these diseases (the financial cost within the United States has surpassed that of cancer and heart diseases^3^), few treatment options exist, and no cures are currently available.

While vascular dementia (VaD) de facto results from vascular injury,^4^ recent evidence has also implicated the role of cardiovascular factors in the progression of AD. Notably, ≈80% of patients diagnosed with AD exhibit vascular pathology when inspected at autopsy.^5^ Other studies have shown altered blood-brain barrier integrity,^6^ alterations in cerebral blood flow,^7^ increased blood pressure,^8^ and increased cerebrovascular resistance.^9^ It is also of note that in preclinical AD, detectable changes in vasculature occur before the detection of current standard AD biomarkers, β-amyloid and tau.^10^ In line with these observations, the use of blood pressure medication by hypertensive patients has been associated with a reduction of dementia risk.^11^ While there are likely multiple routes by which the onset of dementia occurs, interventions targeting a reduction of cardiovascular risk represent thus far one of the most viable and promising strategies for preventing dementia onset or progression.^12^ This is exemplified by the declining incidence rate of dementia within industrialized nations over the last few decades, suggested to be a result at least partially of the successful long-term treatment of cardiovascular disease (CVD).^13^ Despite the strong links between CVD risk and dementia, few studies have examined longitudinal CVD risk in relation to cognitive decline and dementia.

The Framingham Risk Score (FRS) is a well-validated, multivariable risk function used to quantify the risk of a CVD event occurring within a subsequent 10-year period.^14^ This score has previously been found to be a significant predictor of late-life cognitive decline^15-20^ and dementia.^21^ Previous investigations have typically assessed dementia incidence or cognitive decline as a function of cross-sectional FRS value.^21-23^ Such an approach may underestimate the role of CVD risk for cognitive aging: CVD risk increases with age, and there may be large individual differences in the accumulation of risk factors over time.^12^ Following the accumulated change of vascular risk is of particular importance because it more closely captures the ongoing, summative nature of vascular risk development. Understanding the impact of an evolving temporal CVD risk could help guide clinical practice by elaborating the potential for mitigation of dementia risk via interventions directed at CVD risk. Recent research has shown that a longitudinal worsening of CVD risk is associated with midlife cognitive decline,^24^ and the worsening of single CVD risk factors has been shown to be associated with an increased dementia risk.^25,26^ However, few studies have examined, or are equipped to examine, the relationship of longitudinal changes in multiple CVD risk factors with regard to cognitive variation and dementia. Consequently, the current study aims to provide an understanding of the impact of longitudinal, dynamic CVD risk for both dementia incidence and episodic memory (EM) decline. This is achieved by using data from the Betula study, a longitudinal, multicohort study of memory, aging, and dementia. CVD risk, dementia status, and memory performance were measured at 5-year time points across 20 to 25 years in a large sample of healthy adults (n = 1,244; age 35–80 years at baseline). We hypothesized that the longitudinal, accumulated trajectory of CVD risk, as determined by the FRS, can be divisible across the sample and that such divisions will show differences in cognitive decline and dementia risk. Specifically, we hypothesized that individuals with an accelerated CVD risk will have a higher incidence of dementia at older ages and a higher incidence of EM decline in midlife, before the onset of dementia.

## Methods

### Study Design and Participants

**Figure 1:**
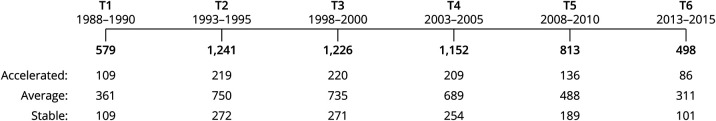
Overview of Sample Size Over Test Sessions and Number of Participants Characterized as Having Accelerated, Average, or Stable FRS Trajectories FRS = Framingham Risk Score.

The data reported in the present study were obtained from the longitudinal, population-based Betula study, based in Umeå, northern Sweden.^27^ Two samples, randomly sampled from the population registry and recruited at 2 different time points (T), were included (T1: 1988–1990; T2: 1993–1995). Data were collected from participants in both samples at 5-year intervals across 25 and 20 years, respectively (Figure 1). At recruitment, each sample consisted of 1,000 individuals each, with 10 groups of 100 participants evenly distributed across age ranges (35–80 in sample 1 and 40–85 in sample 3) and even sex distributions (detailed sample characteristics are shown in Table 1 and provided in full elsewhere^27^). A total of 1,244 participants who had sufficient data and attended at least 2 time points were included in the current work.

**Table 1:**
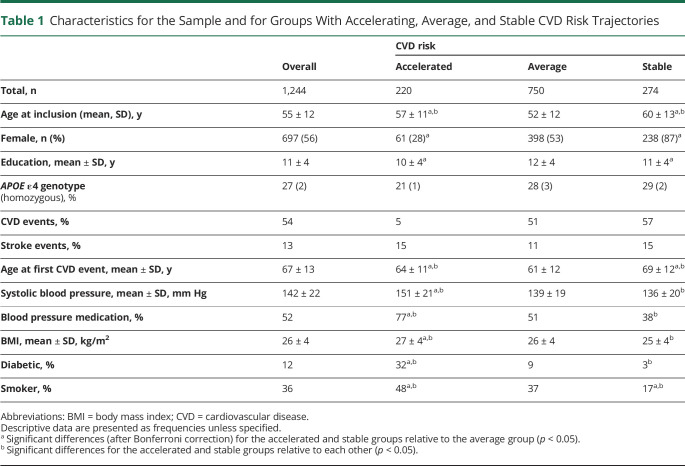
Characteristics for the Sample and for Groups With Accelerating, Average, and Stable CVD Risk Trajectories

### Standard Protocol Approvals, Registrations, and Patient Consents

The Regional Ethical Vetting Board at Umeå University approved this study, and written informed consent was obtained for all participants.

### Health and Cognitive Testing

At each 5-year test session, participants underwent cognitive testing, health examinations (e.g., blood pressure measurements and collection of blood samples), interviews, and completed lifestyle questionnaires. All tests were carried out or guided by a licensed nurse. *APOE* ε4 carrier status was determined via PCR and dichotomized into carriers/noncarriers of the ε4 allele.^27^

Dementia diagnoses were determined through evaluation of written and computerized medical records covering the time span from recruitment to the end of the study period. Diagnoses were clinical and based on the DSM-IV criteria^28^; diagnosis of AD and/or VaD was therefore determined via evaluation of what was considered the primary pathology underlying the cognitive impairment. Medical, psychiatric, and pharmacologic data from inpatient and outpatient care were integrated, when available, in the dementia diagnosis process. Brain imaging may have been available as part of a participant's external clinical assessments but was not implemented within the study as a direct source of information from which to guide the diagnosis procedure. Participants who were found to have dementia at either the time of recruitment or the first diagnostic follow-up were excluded from the study. To increase diagnostic precision, follow-up assessments with the computerized medical record system were performed without the study physician having access to previously determined status with regard to dementia status, subtype, and disease onset. In addition to the medical records, the results from health and memory assessments were considered with regard to the diagnosis. Participants fulfilling 1 or several of the following criteria were considered high risk and underwent more extensive evaluation: (1) low score on a composite cognition and memory tests (≥1.8 SDs below age-based norms), (2) suspected dementia signs observed by the staff conducting the health assessments and cognitive testing, (3) Mini-Mental State Examination score <24 or a decline of at least 3 points in the Mini-Mental State Examination score relative to the prior testing occasion, or (4) a subjective sense of memory impairment reported by the participant. Disease onset was defined as the time at which the clinical symptoms became sufficiently severe to interfere with social functioning and activities of daily living. Individuals with cardiovascular burden accompanied by neurologic signs and a fluctuating course were diagnosed as having VaD. In some instances, a mixed condition was evident; these cases were denoted dementia not otherwise specified and were excluded from the analysis (n = 11 at T6). The diagnosed AD dementia and VaD cases all showed a progressive decline as evident by symptoms attributable to AD dementia or VaD, respectively. Furthermore, patients exhibiting an unspecified condition or exhibiting long-term low cognitive capacity after trauma, stroke, tumor, or subarachnoid hemorrhage were excluded from the analysis.

An EM composite score was constructed from performance on 5 tasks: (1) immediate free recall of visually and orally presented short sentences, (2) delayed cued recall of nouns from the previously presented sentences, (3) immediate free recall of enacted sentences, (4) delayed cued recall of nouns from the enacted sentences, and (5) immediate free recall of a list of orally presented nouns. Each test has been described in detail previously.^29^ Composite scores were constructed by summation of performance on each task. The resulting scores ranged from 0 to 76 (mean 35, SD 12), with a higher score indicating better EM performance.

### Cardiovascular Risk Trajectory Groups

**Figure 2:**
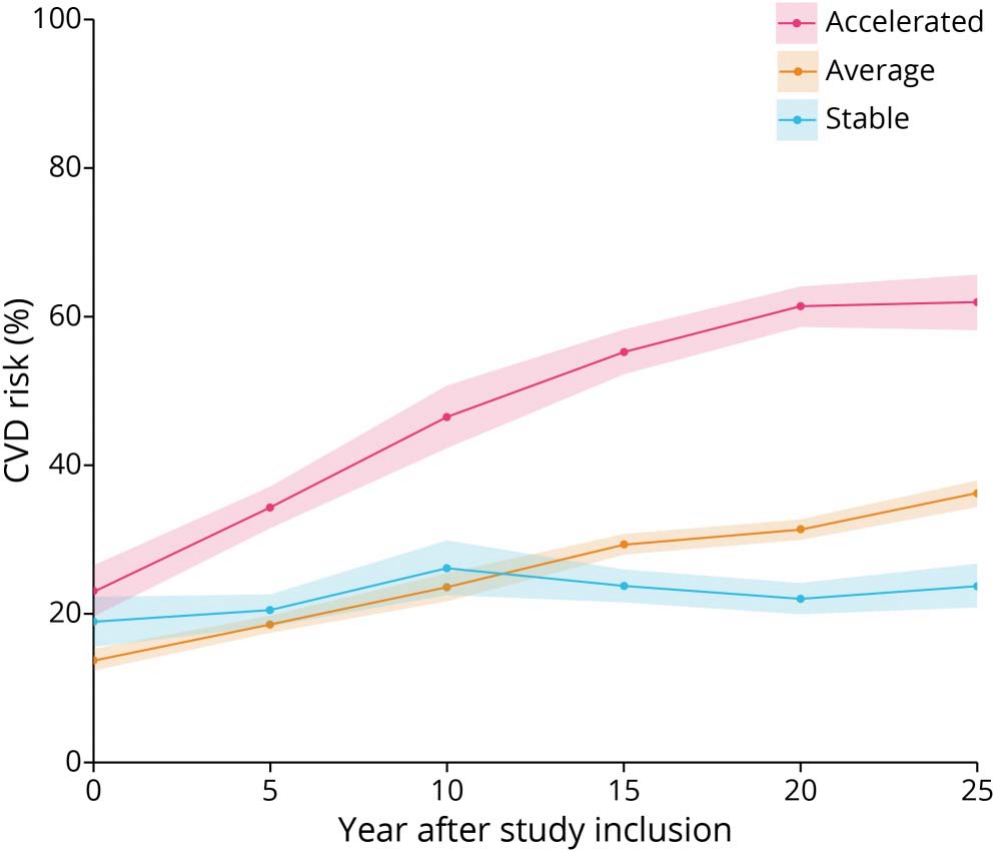
CVD Risk (Percent Within a 10-Year Period) at Each 5-Year Assessment Over 25 Years for Groups With Accelerated, Average, and Stable CVD Risk Trajectories Shaded areas represent 95% credible intervals. CVD = cardiovascular disease.

CVD risk was assessed via the FRS,^14^ which is a tool widely used in clinical settings to predict the likelihood of adverse CVD events. We used the office-based version of the score, which is computed from age, sex, systolic blood pressure, blood pressure medication usage, body mass index, smoking status, and diabetes diagnosis. These factors were aggregated into a multivariable CVD risk score per individual according to earlier descriptions^14^ that represents a probability of having any form of CVD event within a 10-year period. Risk scores were calculated at every time point. The average rate of CVD change over time was analyzed with a linear model based on the FRS values of up to 5 repeated time points over 20 to 25 years while accounting for nonignorable dropout with a pattern mixture modeling approach.^30^ Participants with ≥2 FRS values were classified as having average, accelerated, or stable CVD risk trajectories in relation to their age and individual baseline risk. Accelerated and stable CVD risks were defined as rates of change >1 or <1 SD from the mean, respectively. The average group showed a steady increase in FRS values, reflecting the typical progression of CVD risk. For individuals diagnosed with dementia, only FRS scores before diagnosis were considered. On a group level, each FRS group had similar average starting values (Figure 2).

### Statistical Analysis

Bayesian additive regression tree (BART^31^) is a bayesian machine learning method that combines ensemble learning and semiparametric regression. BART can be implemented for a variety of outcomes, including continuous, binary, and time to event with right censoring. In this work, BART is used to estimate multistate models, to impute missing covariates in the FRS, and to estimate EM decline.

BART was first used to impute the missing covariates in the FRS score, assumed to be missing at random,^32^ with the approach described previously.^33^ Details of the procedure can be found in the eMethods (links.lww.com/WNL/B894).

To characterize the association between dementia, mortality, and CVD risk, we consider 2 multistate models.^34^ The first model is the illness-death model in which each individual starts in the healthy state and, depending on event, transitions to either a state of dementia (AD dementia or VaD) or the final state, death. An individual first diagnosed with dementia can also transition to the final state of death. In the second model, AD dementia and VaD were treated as 2 separate states. The multistate model can be decoupled into a set of survival models, fitting separate intensities to all permitted transitions with the use of BART while making appropriate adjustments to the risk set.^35^ BART was chosen because of its performance as a flexible framework capable of modeling complex, nonlinear, and interaction relationships of covariates when predicting or explaining survival time (for a more complete primer on this methodology, see reference 36). This extends to a capability in handling nonproportional hazards (a requirement for other models, including Cox regression analysis). Furthermore, BART has been found to be more accurate than propensity score matching, weighting, or regression adjustment in nonlinear scenarios (such as in the current analysis^37^).

BART is used mainly for making predictions and does not provide coefficient estimates. To overcome this, we implement a bayesian version of regression standardization^38^ to study the association between CVD risk and dementia risk. This involves estimating the BART survival models while adjusting for covariates (age, sex, education, and *APOE* ε4 status) and the FRS groups. When the model has been fitted, we use Monte Carlo sampling and regression standardization to estimate marginal measures of association, i.e., sample pseudodata (of size 10,000) for the baseline confounders for each posterior sample of the parameters (using the approach described in reference 39). Then, we use the BART model to predict the survival function for the pseudodata separately for each FRS group (treating FRS grouping as fixed). After this, we average these predictions to produce standardized survival functions for each posterior sample. Last, the standardized survival functions for the FRS groups are contrasted to produce standardized (or marginal) measures of association, as though the covariate distribution was the same in the groups. These standardized measures are then used to provide incidence rates for the events that occur (i.e., death, AD dementia, VaD). The relationships of the incidence rates across differing CVD risk trajectory groups are used to provide risk ratios (RRs). A similar procedure is implemented to obtain standardized measures for *APOE* ε4, sex, and age groups whereby we instead standardize over the observed distribution of the sample conditioning for that covariate. Because dementia affects primarily older adults, analyses of dementia incidence were performed on individuals who entered the study at ≥70 years of age (n = 243). Similarly, because we aimed to capture cognitive changes that occur before the onset of dementia, the participants <70 years of age at study inclusion were analyzed for EM changes. Samples from 1,000 of the standardized survival functions were used to obtain the target posterior distribution and to ensure negligible Monte Carlo error. The chains were carefully monitored for convergence and mixing via trace plots.

Implementation of BART for the analysis of cross-sectional FRS values and for the prediction of EM decline is described in the eMethods (links.lww.com/WNL/B894). Analyses were conducted with R version 3.6.3 (R Foundation for Statistical Computing, Vienna, Austria).

### Data Availability

Access to these original data is available on request from the corresponding author and after approval by the Steering Group of the Betula project (umu.se/en/betula).

## Results

### AD and VaD Across Different CVD Risk Groups

Seventy-eight individuals (6.3% of the sample) developed AD dementia, and 39 (3.1%) developed VaD within the 20- to 25-year study time span. In older groups (≥70 years of age at inclusion, n = 243), 32% developed AD dementia and 16% developed VaD. Only participants >65 years of age developed dementia within this cohort. For AD dementia, the absolute risk reduction (ARR) across the whole sample was 2.7% for those in the average group relative to those in the accelerated group, with a number needed to treat (NNT) of 37. Furthermore, the ARR was 2.5% for those in the stable group relative to those in the accelerated group, with an NNT of 40. For VaD, also across the whole sample, both the average and stable groups had an ARR of 3.4% compared to the accelerated group and an NNT of 30 also for both comparisons. At T3 (the point at which all participants recruited at T1 or T2 were asked to return), 1.5% of the sample dropped out. This was followed by 6% of the remaining sample at T4, 30% at T5, and 39% at T6.

**Figure 3:**
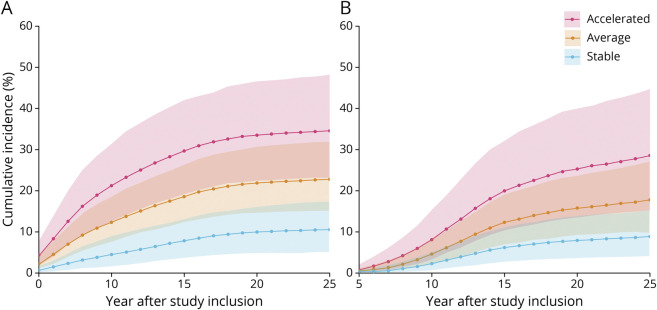
AD and VaD Incidence Across 25 Years in Older Individuals With Differing CVD Risk Trajectories Higher incidence of Alzheimer disease (AD) (A) and vascular dementia (VaD) (B) across 25 years in older individuals (≥70 years of age at study inclusion) with accelerated (n = 57) compared to stable (n = 92) and average (n = 94) cardiovascular disease risk trajectories. Shaded areas represent 95% credible intervals.

Findings from the multistate model show that older adults who had a subsequently stable CVD risk trajectory from study inclusion had a reduced likelihood of developing AD dementia or VaD during the study period relative to participants with an accelerated CVD risk trajectory (Figure 3). Detailed RRs are presented in Table 2. The RR for those in the accelerated group relative to the stable group ranged, on average, from 5.7 to 3.3 across the study time span for AD dementia (95% CI 2.6–17.5 at T2, 1.9–6.7 at T5; eFigure 1, links.lww.com/WNL/B894) and from 4.1 to 3.3 across the study time span for VaD (95% CI 1.1–16.6 at T2, 1.5–7.6 at T5; eFigure 2). Furthermore, those with an average trajectory compared to those with a stable trajectory were more likely to develop AD dementia, with an RR ranging from 3.1 to 2.2 across the study time span (95% CI 1.3–8.9 at T2, 1.2–4.4 at T5). For VaD, the RR for participants with an average trajectory relative to a stable trajectory ranged from 2.1 to 2.0 across the study time span (95% CI 0.7–5.8 at T2, 1.0–4.2 at T5).

**Table 2:**
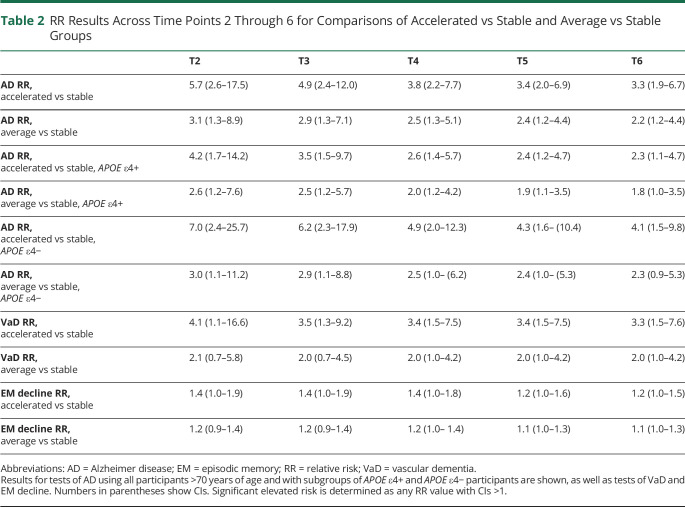
RR Results Across Time Points 2 Through 6 for Comparisons of Accelerated vs Stable and Average vs Stable Groups

Groupings of participants were also made and compared according to FRS cutoffs of <6%, 6% to 20%, and >20%. No associations were found in relation to AD dementia with this approach. Predictions of VaD outcome using baseline cutoffs were revealed to be significant, with the high-risk group (>20%) showing an increased incidence relative to the medium-risk group (6%–20%) from T3 onward, with an average RR of 2.4 at T3 and 2.2 at T5 (95% CI 1.0–7.2 at T3, 1.0–5.8 at T5; eFigure 3, links.lww.com/WNL/B894). The same split using the last available FRS values (i.e., 5 years before a diagnosis) was not found to be a significant predictor of AD dementia or VaD. No other significant differences were found in comparisons of FRS groups split by cutoffs at either baseline or with data from the last available time point. Similarly, comparisons of groups split by tertile or quartile risk level did not reveal any significant differences.

**Figure 4:**
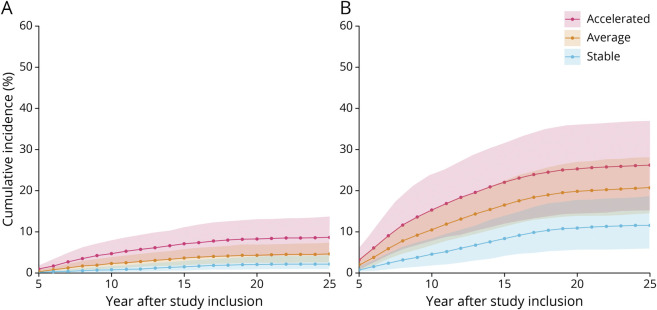
AD Incidence for APOE e4− and APOE e4+ Participants With Differing CVD Risk Trajectories Incidence of Alzheimer disease (AD) is elevated in (A) *APOE* ε4− (accelerated n = 39, average n = 62, stable n = 74) and (B) *APOE* ε4+ participants (accelerated n = 18, average n = 32, stable n = 18) and highest in individuals with accelerated cardiovascular disease risk trajectories. Shaded areas represent 95% credible intervals.

Due to the increased risk of AD dementia for *APOE* ε4 carriers, we assessed potential interactions of carrier status with regard to CVD risk trajectory (Figure 4). Both *APOE* ε4+ and *APOE* ε4− participants who had a stable CVD risk trajectory exhibited a reduced risk of developing AD dementia relative to groups with accelerated trajectories, yet the risk was inflated across all *APOE* ε4+ participants. For *APOE* ε4+ carriers, the RR for those in the accelerated group relative to the stable group was consistently elevated and ranged from 4.2 to 2.3 across the study time span (95% CI 1.7–14.2 at T2, 1.1–4.7 at T5). Similarly, the RR ranged from 2.6 to 1.8 for the average group relative to the stable group (95% CI 1.2–7.6 at T2, 1.0–3.5 at T5; eFigure 4, links.lww.com/WNL/B894). The RRs for the average and accelerated *APOE* ε4+ participants were significantly elevated relative to all *APOE* ε4− participants, presumably as a result of elevated genetic and vascular risk. Average *APOE* ε4+ participants compared to accelerated *APOE* ε4− participants had an RR of 2.3 to 2.5 (95% CI 1.1–5.5 at T2, 1.4–5.2 at T5), while accelerated *APOE* ε4+ compared to accelerated *APOE* ε4− participants showed an RR of 5.0 to 4.7 (95% CI 2.7–10.1 at T2, 2.8–8.8 at T5). However, the stable *APOE* ε4+ participants did not show a significantly increased risk relative to the accelerated *APOE* ε4− participants (RR of 0.9–1.3, 95% CI 0.3–2.5 at T2, 0.6–3.0 at T5), suggesting that a degree of mitigation occurred for those *APOE* ε4+ participants. For *APOE* ε4− participants, the RR for those in the accelerated group relative to the stable group ranged on average from 7.0 to 4.1 across the study time span (95% CI 2.4–25.7 at T2, 1.5–9.8 at T5), while the RR for the average group ranged from 3.0 to 2.3 relative to the stable group across the study time span (95% CI 1.1–11.2 at T2, 0.9–5.3 at T5; eFigure 5).

### Survival Across Different CVD Risk Trajectory Groups

Because death is a competing risk for outcome in the elderly, we also set out to investigate whether FRS trajectory is associated with survival either after or in the absence of dementia (eFigure 6, links.lww.com/WNL/B894). In total, 142 (11.2%) participants died within the study time span (40% of adults >70 years of age at study inclusion). CVD risk trajectory group was not found to be predictive of survival either in the absence of dementia or after AD dementia. For VaD, however, an accelerated CVD risk trajectory, relative to those with an average trajectory, was found to predict a decreased rate of survival after diagnosis, with an RR of 5.1 to 2.7 across the study time span (95% CI 1.6–22.3 at year 0, 1.3–8.7 at year 12).

### CVD Risk Trajectory Is Associated With Early Manifestations of EM Decline

**Figure 5:**
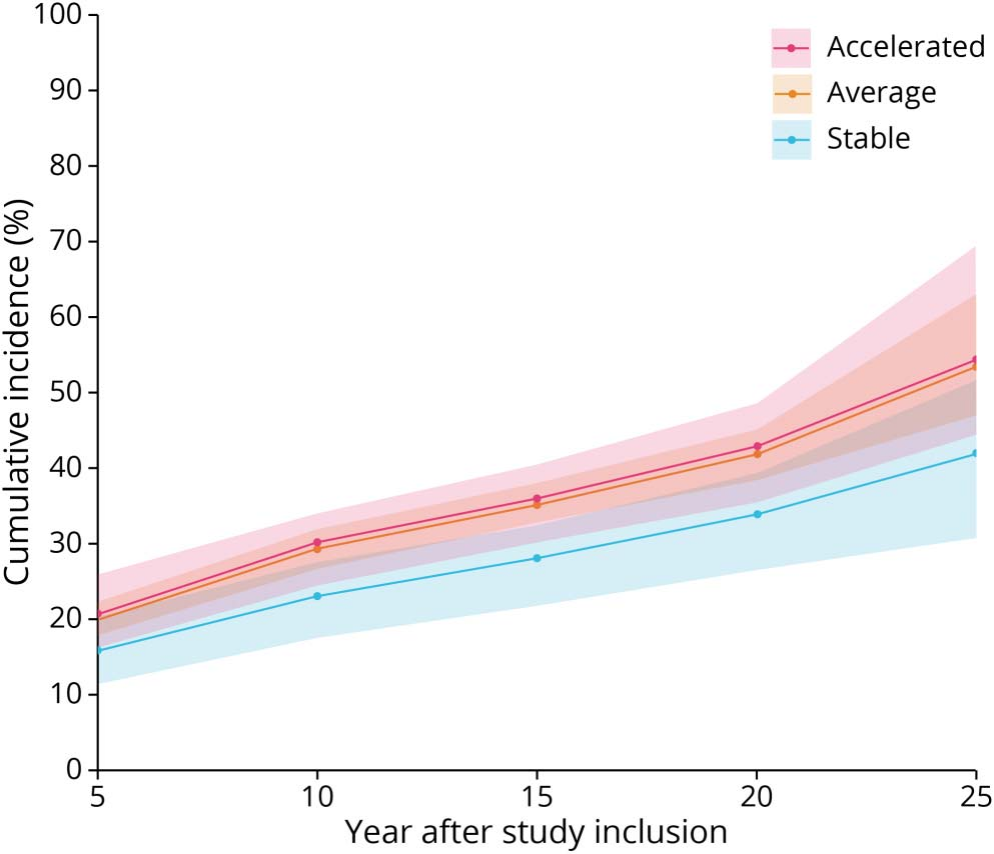
Higher Incidence of EM Decline Events Across 25 Years in Younger Individuals (Aged 35–65 at Study Inclusion) With Accelerated Compared to Stable CVD Risk Trajectories Accelerated n = 163, average n = 656, stable n = 182. Shaded areas represent 95% credible intervals. CVD = cardiovascular disease; EM = episodic memory.

In total, 379 individuals (29.9% of the total sample) exhibited relative EM decline during the observation period. Analysis was carried out on those ≤65 years of age (i.e., in the cohorts <70 years of age) at study inclusion. The risk for EM decline was increased in individuals with an accelerated CVD risk trajectory relative to those with a stable risk trajectory (Figure 5), with an RR ranging from 1.4 to 1.2 across the study time span (95% CI 1.0–1.9 at T2, 1.0–1.5 at T5). The RR ranged from 1.2 to 1.1 for the average group relative to the stable group (95% CI 0.9–1.4 at T2, 1.0–1.3 at T5; eFigure 7, links.lww.com/WNL/B894).

## Discussion

The findings of the current study suggest that the relative trajectory of CVD risk is a significant component in determining the risk of developing dementia in late life and is associated with midlife EM decline in a healthy sample at inclusion. Our findings are complementary to previous research showing an association between longitudinal CVD risk and pathologic cognitive decline yet provide a more dynamic view of this process via a contrasting methodologic approach.^21-23^ The study classifies temporal CVD risk using an analysis of longitudinal data, and links it to both cognitive decline and dementia incidence within the same cohort. These results point to the significance of accumulated vascular risk in relation to healthy brain aging and promote the importance of continual CVD risk observation to allow mitigation via medical treatment or interventions focused on increasing cardiovascular health.^40^

The FRS is well validated and easily accessed^14^ and has for this reason been widely used in research and in clinical practice to predict future CVD risk. Several risk parameters were elevated for the group with an accelerating CVD risk, indicating that such acceleration may arise from an accumulation of damage from a combination of risk factors over time.^41^ While the degree of influence of single risk factors on dementia risk may vary across individuals, addressing a combination of modifiable factors is currently suggested to be the best approach for mitigating or preventing the onset of dementia.^42^ Therefore, assessment of CVD profiles, rather than single risk factors, is encouraged to capture a comprehensive state of CVD-related dementia risk.^17,23,24^

FRS values are elevated by aging alone; hence, active suppression of the modifiable variables of the FRS (i.e., reduction of blood pressure or body mass index or smoking cessation) would have to take place to maintain a stable CVD risk trajectory. Furthermore, because the cross-sectional FRS values offered less predictive utility for survival or dementia outcomes, it is suggested that the relative accrual of multiple risk factors plays a larger part in determining outcome than standalone measurements. An interesting finding is that, despite the strong role of *APOE* ε4 *in* AD risk and the relatively increased proportion of *APOE* ε4+ participants in the stable CVD risk group compared to the accelerated risk group, the proportion of participants developing AD was still significantly lower in the stable CVD risk group. This points toward the importance of maintaining a stable CVD risk even in the presence of increased genetic risk.

The pathologic processes culminating in dementia take place across several years before symptomatic manifestation (such as EM decline).^43^ Of the multitude of factors likely modulating this process, vascular changes are among the first^10^ and have consistently been linked to cognitive decline across the lifespan. EM decline, while showing marked interindividual variation in the rate and pattern of change, is strongly associated with later AD incidence.^44^ In particular, early and rapidly declining EM performance is associated with the transition to an AD state.^30^ Similar EM deficits are found in individuals with VaD; however, the degree of impairment is generally lower than in AD.^45^ The FRS trajectories within the current study are shown to predict the transition from healthy to a dementia state and are associated with EM decline events at earlier ages. This suggests a chain of events whereby increased CVD risk leads to EM decline and ultimately dementia.

Limitations of the current research include the potential for misclassification of AD and VaD cases. While the diagnosis procedure used all resources available within the study framework (detailed above), this did not include neuroimaging or biomarker assessment as part of the standard diagnostic procedure. As a result, diagnoses of AD and VaD reflect the primary pathology as defined per the DSM-IV criteria and are not necessarily made with absolute certainty. In light of this, the RRs for both investigated forms of dementia, while significantly increased in a similar manner, should be treated with caution. However, given the similarity in results for AD and VaD, our findings likely present a generalized increased risk for all-cause dementia. Limitations also include the inability to firmly determine whether the sequence of EM decline leading to dementia is initiated by an accelerated FRS trajectory. Furthermore, while the current study focuses on the involvement of cardiovascular risk in the emergence of cognitive decline and dementia, we cannot rule out that other factors can influence the outcome. For example, tau deposition in the brain has recently been highlighted as a potential mediator of brain atrophy and cognitive decline related to vascular risk.^46^ Because the current study was not able to incorporate data regarding protein deposition (such as β-amyloid or tau), we are not able to entirely exclude the possibility of such a mediating factor. However, previous research has thus far not been able to establish a causal direction of such mediating factors. Furthermore, a large data-driven analysis has suggested that vascular factors influencing AD risk arise before the emergence of abnormal protein accumulation, raising the possibility that the apparent mediation arises as a result of early vascular risk.^10^ While the exact mechanisms underlying a potential association between early vascular risk and tau or β-amyloid deposition remain to be elucidated, both cerebral hypoperfusion and blood-brain barrier degradation have been found to be associated with vascular risk factors (e.g., hypertension, diabetes, and obesity) and to emerge independently of tau biomarker abnormalities in patients with AD.^47,48^

Previous research implicates the importance of maintaining vascular health throughout life as a protective measure against both emergent dementia and cognitive decline, particularly with regard to mechanistic damage (e.g., lesions, infarcts, microbleeds, and reduced blood-brain barrier integrity), which can be irreversible and can elicit further damage.^6-9,49^ Hippocampal integrity (which is degraded within AD) has been shown to be particularly sensitive to vascular stress and critical for EM function.^49^ Such a mechanism may thus serve as a potential mediator of CVD risk and cognitive decline, with vascular stress negatively affecting brain reserve, leading to variable cognitive outcomes downstream of that. The link between low CVD risk and preserved EM may reflect increased brain maintenance within that subgroup, manifested as reduced vascular neuropathology such as fewer lesions and lower perfusion.^50^

Our results indicate that cardiovascular-associated dementia risk likely results from the dynamic progression of combinatorial effects rather than static risk factors. Future research may benefit from further exploring how longitudinal CVD risk affects the accumulation of neuropathology.

## Glossary

AD: Alzheimer disease
ARR: absolute risk reduction
BART: bayesian additive regression tree
CVD: cardiovascular disease
DSM-IV: *Diagnostic and Statistical Manual of Mental Disorders, 4th edition*
EM: episodic memory
FRS: Framingham Risk Score
NNT: number needed to treat
RR: risk ratio
VaD: vascular dementia
